# Recent advances in nuclear actin research

**DOI:** 10.1080/19491034.2025.2498643

**Published:** 2025-05-04

**Authors:** Anikó Szabó, Péter Borkúti, Zoltán Kovács, Ildikó Kristó, Péter Vilmos

**Affiliations:** HUN-REN Biological Research Centre, Institute of Genetics, Szeged, Hungary

**Keywords:** Actin, nucleus, transcription, DNA repair, replication, chromatin, phase separation, nuclear transport, development, disease

## Abstract

Actin was first observed in the nucleus more than sixty years ago but research on nuclear actin did not receive significant attention for the next forty years. It only started to accelerate around the year 2000, when the first convincing experimental data emerged indicating that actin participates in essential nuclear processes. Today, we know that actin is involved in transcription, replication, DNA repair, chromatin remodeling, and participates in the determination of nuclear shape and size. In this paper we review the results of the last five years of increasingly intensive research on nuclear actin, because on one hand, the field has expanded with several new directions during this time, and on the other hand, the enrichment of our picture of nuclear actin will certainly provide a more solid foundation and new impetus for its future investigation.

## Introduction

Actin is one of the most abundant proteins and is evolutionarily highly conserved across eukaryotes. Moreover, it is not only present in eukaryotes but its earliest form likely emerged in the common ancestor of bacteria, archaea, and eukarya [1,2]. Since its discovery, eukaryotic actin was believed to be confined to the cytoplasm, where it primarily contributes to cell shape, movement, and division. However, based on work over the past 25 years, it is now clear that actin is present in the nucleus and participates in basic nuclear functions. Thanks to recent technological advances in actin labeling tools, live imaging and analysis, and functional genomics technologies, the number of laboratories working on nuclear actin, and our knowledge, has grown. Therefore, in this paper, we have undertaken to provide an overview of the new results that have emerged over the last five years preceding the writing of this paper on the role of actin in the three basic functions of the nucleus: transcription, DNA repair and replication, as well as the underlying mechanisms: chromatin remodeling and genome organization. It is important to note that although these are separate processes, there is significant functional overlap. In addition, future research directions will be also discussed, such as the role of actin in nuclear phase separation and transport, and the impact of nuclear actin activity on development and human disease. The final part of the paper discusses recent technical developments related to nuclear actin research. We refer to experimental results published before 2019 at the beginning of each topic with relevant review articles, published preferably in 2019 or before.

## Transcription

**Figure 1.**
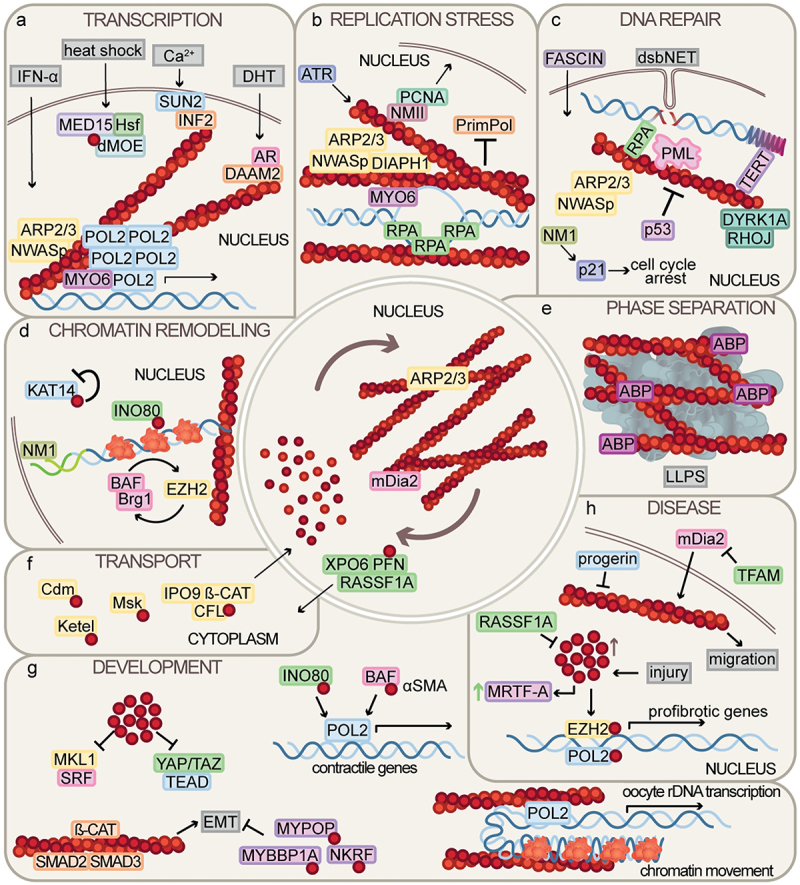
A comprehensive summary of the newly discovered, diverse functions of nuclear actin. (a) Nuclear actin polymerization has been shown to be crucial for Pol2 clustering in response to different stimuli. Pol2 - RNA polymerase 2, AR – Androgen receptor, DHT – Dihydro-testosterone, INF2 - Inverted formin 2, SUN2 - SUN domain-containing protein 2, MED15 - Mediator of RNA polymerase II transcription subunit 15, Hsf – Heat shock factor protein, dMoe – *Drosophila* Moesin, DAAM2 - Disheveled-associated activator of morphogenesis 2, ARP2/3 - Actin-related protein 2/3 complex, nWasp - Neural Wiskott-Aldrich syndrome protein, MYO6 – Myosin VI. (b) Local actin polymerization acts as a framework for replication fork stalling and repair in the event of replication stress. ATR – ataxia telangiectasia and Rad3-related protein, PCNA – Proliferating cell nuclear antigen, NMII – Non-muscle myosin II, PrimPol – DNA-directed primase/polymerase protein, DIAPH1 - Diaphanous related formin 1, RPA – Replication protein A. (c) Nuclear actin fibers are required for chromatin and PML body movement, repair factor clustering and limiting access of other factors upon DNA damage. dsbNET – DSB-capturing nuclear envelope tubules, RPA – Replication protein A, TERT – Telomerase reverse transcriptase, PML – Promyelocytic leukemia nuclear body, p53 - Cellular tumor antigen p53, DYRK1A – Dual specificity tyrosine-phosphorylation-regulated kinase 1A, RHOJ – Rho-related GTP-binding protein, NM1 - Nuclear Myosin 1, p21 - Cyclin-dependent kinase inhibitor 1. (d) Monomeric actin acts as a component of chromatin remodeling complexes, while filamentous actin provides a scaffold for chromatin movement. KAT14 - Cysteine-rich protein 2-binding protein, INO80 - Chromatin-remodeling ATPase INO80, BAF – BRG1/BRM-associated factor complex, BRG1 - Brahma homolog 1, EZH2 - Enhancer of Zeste homolog 2, green DNA segment – *Hsp70* locus. (e) Several actin-binding proteins (ABP) contain disordered protein regions, possibly contributing to phase separation involving actin, not only in the cytoplasm, but also in the nucleus. LLPS – liquid-liquid phase separation.(f) the nuclear transport of actin is assured by multiple factors. Cdm – Cadmus, Msk – Moleskin, PFN – Profilin, CFL – Cofilin, IPO9 – Importin 9, ß-CAT - ß-Catenin, XPO6 – Exportin 6. (g) Through its role in transcription and chromatin remodeling, actin is part of several developmental pathways. MKL1 - Myocardin-like protein 1/MRTF-A, SRF – Serum response factor, YAP/TAZ – Yes-associated protein/WW domain-containing transcription regulator protein 1, TEAD – TEA domain transcription factor, MYPOP – Myb-related transcription factor, partner of profilin, MYBBP1A – Myb-binding protein 1A, NKRF – NF-kappa-B-repressing factor, EMT – epithelial-mesenchymal transition. (h) Both the monomer and polymer forms of actin are factors in pathogenic processes, such as migration of cancer cells and response to injury. mDia2 - Diaphanous-related formin protein 2, TFAM – Mitocondrial transcription factor A, RASSF1A – Ras association domain-containing protein 1, MRTF-A - Myocardin-related transcription factor A. The actin filament is symbolized by a double helix made up of round, dark red G-actin monomers, and DNA is symbolized by a blue wavy line. See text for further details.

It has been well established in the first decade of the 2000s that actin plays a critical role in transcription by influencing the organization and dynamics of the transcriptional machinery [3,4]. It directly and functionally interacts with all three RNA polymerases and numerous transcription factors and regulators (Myocardin-related transcription factor A (MRTF-A) [5,6], ß-catenin [7], MED15 [8], Estrogen receptor α [9], Coronin 2A [10]), associates with actively transcribed genes and ribonucleoprotein complexes during transcription, thereby helping to regulate the efficiency and speed of transcription initiation and elongation. For a long time, this ability of actin was attributed primarily to its monomeric form [11], and the idea was also supported when it became clear that transcription factors are controlled by G-actin [12]. Today, however, data discussed below have revealed that the polymeric form of actin is involved in transcription not only through chromatin remodeling and organization but also in the functioning of the gene transcription machinery, in particular during the initiation of transcription and the assembly of the complex (Figure 1(a)).

Actin polymerization in the nucleus is best observed in response to stimuli, and its role in the induced transcriptional response can then be investigated. The presence of factors required for actin polymerization and their activity in the nucleus during these stress responses further supports the idea that polymeric actin is an active participant in nuclear processes. For example, Wang and colleagues reported that Inverted formin 2 (INF2) is required for RNA polymerase 2 (Pol2) cluster formation and nuclear actin assembly upon serum-induced, G-protein-coupled receptor (GPCR)-mediated calcium release [13]. Later, it was found that the serum-induced clustering of Pol2 is not only accompanied by but also dependent on N-WASp/ARP2/3-mediated nuclear actin polymerization, and similar observations have been made for IFN-γ stimulation, suggesting a general role for nuclear actin in Pol2 clustering [14]. Recently, the Grosse lab reported that nuclear F-actin is involved in testosterone-stimulated gene expression [15]. Using super-resolution microscopy, they visualized nuclear microfilaments at the androgen receptor and showed that their assembly is assisted by the DAAM2 formin. This process was shown to drive AR droplet formation upon DHT (dihydro-testosterone) treatment, which colocalize with active Pol2, facilitating the expression of target genes. These results suggest that F-actin is required for clustering with Pol2 to form a transcriptionally active site that ultimately promotes the expression of target genes.

The presence of polymeric actin in the nucleus suggests the use of F-actin-based motility, for which motor proteins, the myosins, are responsible. Their involvement in transcription emerged more than 20 years ago, and the first data on the underlying molecular mechanism were also available more than a decade ago [16]. Overall, eight myosin types have been identified within the nucleus, and research related to nuclear myosin I (NMI) and myosin VI has yielded the most results. Recent studies in embryonic fibroblasts from NMI-knockout mice have shown that NMI is directly involved in DNA damage-induced gene transcription. Accordingly, NMI is enriched at the transcription start site (TSS) of the p21 checkpoint regulator, and occupancy increases upon DNA damage. In this case, NMI forms a complex with p53 and activates p21 expression by recruiting histone acetyltransferases to its promoter [17]. Regarding myosin VI, it was previously known that an autophagy receptor, NDP52 activates myosin VI in the nucleus, and together they bind Pol2 and activate transcription [18]. Recent research has also shown that NDP52 clusters with Pol2 at transcription initiation sites and thereby alters DNA conformation to modulate Pol2 transcription [19], and that myosin VI acts as the molecular anchor that holds Pol2 in high-density clusters [20]. Myosin VI is also present in the nucleolus, but recent data suggest that it is important there for nucleolar organization and ribosome maintenance but not for RNA polymerase 1-related transcription [21].

Among other actin-binding factors, the involvement of the F-actin-binding Ezrin/Radixin/Moesin (ERM) proteins in transcription has only recently become clear. The only representative of the three mammalian ERM proteins in *Drosophila*, Moesin was shown to interact with the Mediator complex, the central integrator and coactivator of transcription, through direct binding to its Med15 subunit [8]. Both Moesin and Med15 bind to heat shock factor (Hsf) and monomeric actin, and together they are required for proper heat shock gene expression under physiological conditions. In the future, it will be of interest to study the monomeric actin binding of nuclear ERM proteins and in particular the role of actin in the function of the transcription regulatory Mediator complex.

Actin is subjected to more than hundred post-translational modifications, including acetylation, oxidation, phosphorylation, and SUMOylation [22]. These modifications likely influence its interactions with other proteins and its functions. Accordingly, yeast actin with an arginine mono-methylation mark on an evolutionarily conserved R256 residue (R256me1) was detected in actin-containing chromatin-modifying complexes INO80, SWR1, and NuA4, and localized to Pol2 and Pol3-transcribed genes [23]. This result explains the long-known observation that the mutations in the corresponding residue, R258, in human α-actin isoforms cause a number of human diseases [24].

## Replication

The recognition that replication and DNA repair require the polymerized form of actin occurred early in the research of nuclear actin. F-actin along with several actin binding proteins (ABPs) mediating and regulating actin polymerization have been found to be crucial for replication fork rescue and the repair of DNA breaks, with special attention to heterochromatin as it is a challenging environment for repair processes [25]. According to the models, nuclear F-actin facilitates the directed movement of double-strand breaks (DSBs) and stalled replication forks toward the nuclear periphery. Recent research is focusing on elucidating the underlying mechanistic role of F-actin in the replication stress response (RSR) and DNA damage response (DDR). Similar to the research on the already well-known cytoplasmic functions of F-actin, the identification of the protein partners that bind and organize nuclear microfilaments is of great help in RSR and DDR research, and is thus a major focus of current research (Figure 1(b)).

Han and colleagues demonstrated that WASp, which mediates actin polymerization, enhances the binding of Replication protein A (RPA) to ssDNA during replication stress and DNA repair through its RPA-binding motif, and that the process also requires N-WASp, DIAPH1 and ARP2/3 [26]. Nuclear actin itself has also been found to interact with RPA, and the polymerization-defective G13R actin mutant exhibits a higher affinity to RPA than the wild type or the S14C hyperpolymerizing mutant. Overexpression of G13R actin caused a similar phenotype to the depletion of nucleation-promoting factors and actin nucleators (NPF/AN), suggesting that actin filament assembly is required for proper RPA loading [26]. These results were confirmed by the finding that NPF/AN family proteins, namely WASp, N-WASp, DIAPH1, and ARP2/3 are recruited to replication forks upon replicative stress, and depletion of either factors significantly reduces average fork velocity and impairs fork restart upon replicative stress induction [27].

Genotoxic stress-induced slowing and reversal of fork growth are also dependent on ARP2/3-mediated nuclear actin branching [28]. Inhibiting actin polymerization by incubating the cells with drugs under these stress conditions induces unrestrained fork progression and discontinuous DNA synthesis by the PrimPol primase-polymerase in the nucleus. The authors suggested that nuclear actin polymerization near replication factories limits the access of PrimPol to ssDNA and allows for the complex process of fork reversal and proper repair of the damage site. The exact mechanism behind is not yet known, but the authors offer several hypotheses. One possibility is that nuclear F-actin enhances chromatin compaction, thereby limiting the access of potentially dangerous replication factors (e.g., PrimPol), which may facilitate a rapid response to replication stress. On the other hand, nuclear microfilaments can also influence fork plasticity by modifying the position and dynamics of replication sites. Finally, it cannot be excluded that actin polymerization indirectly helps to resolve replication stress by reducing the concentration of monomeric actin available to chromatin remodeling complexes at the replication site.

While the results of the previous study were obtained by examining the immediate response to replication stress, the Cesare group studied the effect of prolonged replication stress and found that induction of persistent replication stress in cultured human cells also causes the formation of actin polymers in the S-phase nuclei of treated cells, dependent on ATR kinase [29]. This phenomenon was accompanied by the increase of nuclear volume, presumably to counteract the nuclear deformation induced by replication-stress. PCNA (Proliferating Cell Nuclear Antigen, a replication fork marker) foci were shown to move toward the nuclear boundary along nuclear actin filaments with the help of the motor protein, myosin II. General inhibition of actin polymerization by treating cells with drugs resulted in the suppression of replication fork repair and the appearance of chromosome segregation errors, emphasizing the importance of nuclear actin dynamics in handling replication stress.

The involvement and importance of actin motor proteins, myosins, in replication is a long-known observation, which clearly provides strong support for the role of polymeric actin in replication. Recently, myosin VI was shown to contribute to the stabilization of stalled or reversed replication forks by cooperating with the AAA ATPase Werner helicase interacting protein 1 (WRNIP1) [30]. Both the motor and ubiquitin-binding activity of myosin VI is involved in protecting replication forks from DNA2-mediated nucleolytic attack, indicating that F-actin is involved in the process. Moreover, the codepletion of fork remodelers RAD51, HLTF, SMARCAL1, or ZRANB3 alongside myosin VI prevented nascent strand degradation, suggesting that the fork stability defect caused by the lack of myosin VI relies on the prior activity of the fork remodelers. Therefore, the authors concluded that myosin VI does not inhibit fork reversal but it protects reversed replication forks.

Interestingly, in contrast to stress conditions, actin polymerization was found to be dispensable for replication fork progression under non-stress conditions [28], and accordingly, a small percentage of PCNA foci were observed to be associated with F-actin [31]. However, the number of F-actin associated foci increases even with mild replication stress, presumably representing stalled or broken forks. These foci were determined to be more mobile, suggesting that even without stress or in relatively low-stress conditions, F-actin aids fork relocalization for repair [31]

The question is still open today as to how exactly F-actin, or an actin network, protects the replication fork. One possibility is that the dense filamentous structure, either consisting of actin alone or together with the lamina physically protects the chromatin from mechanical insults through limiting access of proteins. This mechanism may be supported by the phase-separating ability of actin, to be discussed later. Alternatively, actin may perform this function by actively removing harmful components and delivering RSR-competent factors with the help of motor proteins. Since there is experimental support for each of these mechanisms, it is likely that they are all involved in replication fork protection.

## DNA damage repair

Similar to replication, the role of actin in DNA repair has long been largely limited to convincingly demonstrating that actin is involved in the process [32]. It has been clear that an important function of actin is to move DNA during DDR, but recent research has also highlighted that its role is much more diverse (Figure 1(c)). Despite this, our understanding of the mechanistic role of actin in DNA damage repair is still quite limited.

Consistent with previous findings, actin has been implicated also in the dynamics of promyelocytic leukemia (PML) bodies [33], which are part of the DNA damage response and undergo supramolecular fission, after which the resulting microbodies localize to DNA repair sites [34]. Cobb et al. have affirmed that nuclear F-actin formation is required for proper damage response, and demonstrated that PML bodies localize along actin filaments, presumably to facilitate the motility of these nuclear bodies. The authors have also described that expression of prelamin A, a precursor of lamin A abrogates the stress-induced increase in nuclear F-actin and promotes actin relocalization to the nuclear envelope. This disruption of the nuclear actin network was shown to affect PML body number and localization, most significantly attenuating their colocalization with DNA damage sites. Expression of exportin-6, the nuclear export factor of actin caused a similar phenotype, confirming that the changes in PML body dynamics are a result of the disrupted actin network in the nucleus. Accordingly, restoring nuclear actin filaments rescued proper PML function.

Further evidence for the role of nuclear actin in chromatin movement during DDR is provided by the finding that nuclear actin filaments are also involved in localizing DSBs near the nuclear periphery [35]. The authors detailed the formation of DSB-capturing nuclear envelope tubules (dsbNET) that form as a response to DNA damage, and colocalize with actin filaments. Disruption of actin polymerization decreased the appearance of dsbNETs, suggesting that F-actin plays role in guiding the tubules to damage sites. Recent genome-wide analysis in mouse embryonic fibroblasts uncovered that nuclear actin dynamics also affects chromosome rearrangements resulting from DSBs [36]. ARP2/3 and its activator WASP promote homology-directed repair by clustering DSBs, which, however, increases the risk of chromosomal translocations. Upon inhibiting ARP2/3-mediated polymerization of actin in the cell with wiskostatin treatment, the frequency of chromosomal translocation was decreased, further highlighting the importance of actin dynamics during DNA repair.

In the recent past, progress has been made in identifying factors that promote nuclear actin polymerization and localization in DDR. RHOJ, a small Rho GTPase was found to be a regulator of nuclear actin polymerization during DDR. Debaugnies and colleagues have shown that RHOJ, a mediator of replicative stress tolerance that promotes DNA repair in epithelial-mesenchymal transition (EMT) tumor cells, does so in a nuclear actin polymerization-dependent manner [37]. Research has uncovered also that DYRK1A, a serine/threonine kinase and Spir1, an actin nucleator Spire protein are also part of the intricate network of proteins that regulate nuclear F-actin assembly at DSBs, through the phosphorylation of Spir1 by DYRK1A at damage sites [38]. Nuclear actin has been shown to directly bind the Snail family transcriptional repressor 2 (Slug) and stabilize its localization to γH2AX foci during DNA repair in HeLa cells [39]. In experiments aimed at improving doxorubicin chemotherapy, it was found that global inhibition of actin polymerization by Latrunculin B treatment hinders double-strand break repair by impairing the recruitment of replication protein A (RPA) to the site of damage. In addition, actin binders reduce the autophosphorylation of DNA-dependent protein kinase during nonhomologous end joining [40]. Moreover, a recent study identified several compounds that promote or inhibit nuclear F-actin bundling during DDR [41]. The results shed light on the role of an important regulator of F-actin bundling, Fascin, which was found to be actively transported into the nucleus, where it supports F-actin assembly, efficient DDR, and cancer cell survival [41]. It is likely that the direct association of Fascin with phosphorylated histone H3 regulates the protein’s actin bundling activity in the nucleus.

The tumor suppressor p53, the so-called ‘guardian of the genome’, which plays a prominent role as a facilitator of DNA damage repair, has also been shown to influence nuclear actin polymerization. Interestingly, the data suggest that it has a negative regulatory role, as it was found that in human cancer cells loss of p53 promotes the formation of nuclear actin filaments that appear in response to DNA damage [42]. In these cells nuclear actin network did not appear in response to doxorubicin-induced DNA damage alone but was only observed upon concomitant p53 silencing. The authors have revealed that suppression of filament formation is reliant of caspase activation by p53. Increase in the levels of nuclear G-actin was also not sufficient to induce nuclear actin filament formation. Although the authors take into account the side effects of the several actin labeling probes used in their work on actin function and polymerization, the explanation for their observations and the underlying molecular mechanism remains unclear.

The involvement of various actin motor proteins in DDR has long been described, mainly in the translocation of heterochromatic double-strand breaks to the nuclear periphery and in the relocation of chromosomal regions [43]. Today, we only have a significant amount of knowledge about the activity of Myosin I in DDR, and we currently have no information about specific nuclear myosin – DNA damage protein interaction. As shown by recent data discussed above, NMI is indirectly involved in DDR through the initiation of DNA damage-induced transcription [17].

Most recently, nuclear actin has also been implicated in the repair process of telomeres. Harman and colleagues demonstrated that the recruitment of telomerase to telomeres upon the induction of the DNA damage response is also dependent on actin polymerization. In concert with this observation, the global inhibition of key actin regulators mTOR, ARP2/3, WASp, LIMK, myosin, cofilin 1, formin 2 and importin 9 in the cell resulted in decreased presence of telomerase at telomeres [44].

## Chromatin remodeling and organization

All three major nuclear functions discussed above are closely linked to chromatin dynamics and 3D genome structure. Thus, the restructuring of chromatin is an important and strictly regulated process that involves several multi-subunit protein complexes. The presence of actin and actin-like proteins in these complexes, like INO80, SWI/SNF and TIP60 has long been confirmed, but we are just starting to understand their activity, their precise position within the complex, the conditions for their presence, and the significance of all this. Only in the last few years have experimental results been obtained, primarily through the identification of the players involved in the mechanism, which opened the way for us to build the first, relatively complex, and reliable models (Figure 1(d)).

The INO80 complex contains monomeric actin and three actin-related proteins, Arp4, Arp5 and Arp8 [45]. Cryo-EM reconstruction of the yeast INO80 complex have shown that it is quite flexible and has three major conformations, namely open, collapsed and intermediate, that differ in the relative position of the tail domain to the head [46]. The 3D reconstruction has shown that the actin/Arp (actin, Arp4 and Arp8) module is located at the tail of the complex, while Arp5 is part of the head module. To shed light on the mechanism of actin’s involvement, the authors have determined that the actin/Arp module of INO80 is capable of binding to nucleosomes independently of the ATPase domain. They have also suggested that nucleosome binding is initially done by the Arp8 subunit which undergoes a conformational change to facilitate the contact between nucleosome and the other members of the actin/Arp module. Another recent study has shown that the monomer/polymer actin ratio within the cell affects the function of the INO80 complex. Rapid chromosome fragmentation in yeast, which activates glycosylases and endonucleases of the base excision repair (BER) pathway, is accompanied by cytoplasmic actin depolymerization and the parallel accumulation of nuclear actin. Elevated levels of nuclear G-actin was shown to stimulate INO80 subunit C to increase DNA polymerase processivity and convert single-strand lesions into double-strand breaks [47].

It was previously observed that nuclear actin is required for adipogenesis in mouse embryonic fibroblast cells however, it has recently become clear that it achieves this function by maintaining chromatin accessibility at the promoter-proximal region of the CEBPA gene locus that regulates gene expression during transcriptional reprograming of MEFs into adipocytes [48]. At the TSS and the promoter-proximal region of the CEBPA gene, actin maintains low H3K9Me3 level and regulates the genomic deposition of BRG1, the ATPase subunit of the chromatin remodeling complex SWI/SNF. A comprehensive genomic analysis of β-actin knockout mouse embryonic fibroblasts by the Percipalle lab revealed that change in nuclear β-actin levels induces 3D genome architecture reorganization by influencing the interplay between chromatin remodelers BAF/BRG1 and EZH2, the catalytic subunit of Polycomb repressive complex 2, and triggering the accumulation of EZH2 and H3K9Me3 in specific genomic regions [49]. In a later work, they investigated the mechanism by which the change in 3D genome structure due to a decrease in nuclear actin levels leads to the reshaping the gene expression pattern, and concluded that changes in nuclear β-actin levels directly impact the acetylation of H3K27 and result in dramatic alterations in enhancer activity [50].

In addition to ATP-dependent chromatin remodeling complexes, actin also plays an important role in the histone modification complexes. The direct involvement of actin in histone modification as a member of human Ada-Two-A-containing (hATAC) histone-modifying complex was investigated by Viita and colleagues [51]. The authors used affinity purification-mass spectrometry (AP-MS) and proximity-dependent biotin identification-mass spectrometry (BioID-MS) to identify interacting partners of nuclear actin. One of their discoveries was that binding partners of actin and actin-R62D (a mutant form incapable of polymerization) almost completely overlapped, suggesting that polymerization of actin is not required for the interaction with the detected binding partners. One of the novel interactors discovered was KAT14, a member of the hATAC complex. The study has shown that overexpression of NLS-actin or NLS-actin-R62D leads to a decrease in H4K5ac, the target of KAT14, suggesting that monomeric actin negatively affects the HAT activity of KAT14 [51].

While these functions of nuclear actin clearly require its monomeric form, polymerized actin has long been known to be invaluable in moving chromatin [52]. Recently, nuclear actin polymerization was reported to play a role in calcium-induced chromatin remodeling. Ca^2+^ release from the endoplasmic reticulum induced by G-protein coupled receptors promotes the translocation of the formin INF2 to the inner nuclear membrane, where it facilitates the polymerization of nuclear actin filaments. The formation of microfilaments was determined to be required for chromatin reorganization triggered by GPCR/Ca^2+^-signaling [13] (Figure 1(a)). Later, it was demonstrated that calcium-induced nuclear actin assembly and chromatin remodeling requires the co-localization and association of the inner nuclear membrane protein SUN2 with INF2, and that this activity of SUN2 is independent of its function as a component of the LINC complex [53]. The deletion of the Arp subunit components of SWR-C and INO80 remodeling complexes in yeast inhibits the movement of the INO1 gene locus [54] which typically localizes to the nuclear periphery upon inositol induction [55]. Blocking actin polymerization with Latrunculin-A treatment or expressing NLS-actin-R62D had the same effect, suggesting that actin polymerization plays role in INO1 motion. The authors also found that nuclear actin showed dynamic polymerization/depolymerization, and highlighted the role of ABPs in maintaining proper behavior of nuclear actin [54]. The study also sheds light on the involvement of chaperones in regulating the binding between F-actin and Arp-containing remodeling complexes, which in turn contributes to chromosome motion. The role of myosins in chromatin rearrangement has also been confirmed, which also suggests the involvement of polymeric actin. Depletion of NM1 inhibits the heat-shock-induced repositioning of the Hsp70 gene locus away from the nuclear envelope upon heat shock, suggesting that they are involved in the regulation of the spatial dynamics and function of the Hsp70 gene [56]. Recently, nuclear actin and its polymerization have also been shown to be necessary for the maintenance of normal chromatin organization and gene expression in *Arabidopsis* [57], indicating that the phenomenon and the underlying molecular mechanisms are likely widespread among eukaryotes. Most recently, the Rubin laboratory has shown that the global change of the polymerization state of actin in the cell greatly perturbs chromatin landscape [58]. They employed CK666, an ARP2/3 inhibitor which prevents secondary branching of actin polymers, and Cytochalasin D, an actin polymerization inhibitor that induces G-actin transport into the nucleus. Both treatments changed the polymerization state of nuclear actin, which in turn altered chromatin accessibility as demonstrated by ATAC-Seq. In accordance, the increase in free nuclear actin levels also influenced the localization of heterochromatin markers H3K9me3 and H3K27me3. The authors also showed that knockdown of Arp4, a protein known to suppress actin polymerization and bind monomeric actin [59] resulted in changes in chromatin accessibility (mostly increase), noting that Arp4 and potentially its interaction with monomeric actin is also involved in chromatin state regulation. In addition, nuclear actin-mediated changes in H4K5ac levels and chromatin accessibility were also shown to be modulated by the 5-HTR6 (5-hydroxytryptamine receptor 6) signaling pathway in mature neurons in the hippocampus [60].

In addition to the obvious and versatile role of actin in chromatin modification, we have recently learned more about the similar function of actin-like proteins. In addition to the already mentioned ARPs of chromatin remodeling complexes, testis-specific ARPs ACTL7A and ACTL7B have recently been found to be essential for chromatin remodeling during spermiogenesis. *In silico* models predicted that ACTL7A and ACTL7B are capable of binding to the Helicase-SANT-associated (HSA) domain of chromatin remodeling complexes, indicating their role in the process [61]. Utilizing KO mice, the authors discovered that loss of ACTL7B not only results in a shift in transcriptional alterations, including the downregulation of ACTL7A, but also causes the loss of intranuclear lysine acetylation of H3 and faulty localization of HDACs 1 and 3 to the cytoplasm in round spermatids. How these ARPs are involved in the proper nuclear localization of HDACs remains an open question.

## Phase separation

Phase separation is a process in which a homogeneous mixture separates into distinct regions or phases, each with different physical properties. The phenomenon is essential in the functioning of biological systems through the formation of biomolecular condensates that organize membrane-less compartments. These structures are established through liquid–liquid phase separation (LLPS), where biomolecules self-organize into liquid droplets [62].

The concept that actin plays role in LLPS was introduced in the late 2010s [63,64]. In the 2020s detailed investigations began tying LLPS to actin polymerization, uncovering roles of actin-binding proteins in guiding phase-separated systems driving filament assembly [65–68]. The latest studies showcase advances in both cutting-edge biophysical techniques and computational modeling to investigate intricate phase behaviors of actin and their cellular ramifications [68–72]. These studies together led to the realization that actin contributes to the dynamic organization and stability of phase separated structures through its polymerization and interaction with other proteins. In phase-separated condensates, actin filaments can provide a scaffold that helps organize molecular components, and its ability to undergo rapid polymerization and depolymerization allows for the reversible nature of phase separation, enabling cells to respond quickly to changing conditions.

Given that in prokaryotic cells LLPS is the basis for intracellular activities in the absence of an endomembrane system [73], and that the nucleus itself is of prokaryotic cell origin [74] and operates without membrane-bound organelles, functional factories in the nucleus also operate by phase separation [75]. Although direct investigation of the function and regulation of actin in nuclear LLPS, which provides the basis of nuclear bodies, is still awaited, based on the above, it is an obvious assumption that in the nucleus actin plays a role of the same or very similar importance in LLPS to that observed in the cytoplasm (Figure 1(e)). Accordingly, the finding that β-actin depletion can potentially affect the regulation of both HP1α and PRC1 proteins [76,77], which have been implicated in initiating phase separation [78,79], suggests a link between nuclear actin and the formation of phase separated nuclear bodies [49]. Accordingly, the Hozák lab identified MPRIP, an F-actin-binding protein, as a component of the RNA polymerase II/nuclear myosin I complex and showed that in the nucleus MPRIP forms phase-separated condensates that are able to bind F-actin filaments [80]. Two recent bioinformatic studies have further confirmed the role of actin in nuclear LLPS. One study identified 47 actin-binding proteins that localize in the nucleus during heat shock and revealed that the majority of these contain intrinsically disordered regions (IDRs) known to promote LLPS [81]. In the other bioinformatic analysis, the structure of actin-binding proteins was investigated and it was shown that they are mainly intrinsically disordered proteins, many of which are part of membrane-less organelles [82]. Interestingly, it was also found that, although the drivers for the formation of membrane-less organelles are significantly greater in number in the cytoplasm than in the nucleus, the number of intrinsically disordered actin-binding proteins in the nucleus is higher than in the cytoplasm [82].

## Transport

It is well established today that actin is transported through the nuclear pore complex (NPC) as a monomer by active transport. Its import is mediated by a complex of importin-9 with cofilin, whereas its export requires profilin binding and the exportin-6 transporter [83]. Recently, it has also become clear that the direct interaction of Ras association domain family 1 isoform A (RASSF1A) with exportin-6 is necessary for the association of the exportin with the RAN GTPase to export actin monomers in complex with profilin from the nucleus [84] (Figure 1(f)). It has also been known for a long time that the amount of nuclear actin is tightly and dynamically regulated by the cell through modifying its import and export. A recent confirmation of this is provided by the observation that heat stress in *Drosophila* embryos increases cofilin activity, which leads to increased nuclear import rate of free actin and ultimately causes the formation of stable actin rods in the nucleus [85]. An opposite way of regulation has been observed during neuronal differentiation of rat PC6.3 cells. Nuclear actin levels were increased as a result of decreased export rate, most likely due to enhanced retention of actin in nuclear complexes required for differentiation and possibly the downregulation of exportin-6 activity through yet unknown means [86].

It should be noted that despite all these results, the possibility of passive diffusion cannot be completely ruled out due to the size of monomeric actin, and there are data indicating that the mechanisms of active nuclear transport of actin may also show evolutionary divergence. In line with this, recent experiments in *Drosophila* suggest that actin entry into the nucleus is mediated by ways other than the already known importin-9-mediated mechanism, as the amount of actin in the nucleus is still significant even in the complete absence of importin-9 (Figure 1(f)). Accordingly, three additional importins have recently been identified that may be involved in the nuclear transport of actin in *Drosophila* [87]. Interestingly, the piggyback mechanism outlined above for actin entry into the nucleus might exist not only with cofilin but also with other actin-binding proteins, or perhaps occurs in concert with them. Upon applying dynamic mechanical strain on mesenchymal stem cells, the nuclear entry of the actin-binding β-catenin protein was found to be dependent on the nuclear transport of actin, as knockdown of cofilin-1 or importin-9 inhibited the nuclear import not only of actin but also of β-catenin [88]. Because β-catenin has no NLS, the authors hypothesized that its nuclear import depends on the interaction with actin transported into the nucleus through the cofilin/importin-9-dependent mechanism (Figure 1(f)).

The role of actin in the movement of various nuclear components is primarily known in connection with chromatin movements during transcription and DNA damage repair, which has been discussed previously. The transport of RNA and protein molecules in the interchromatin space, according to our current knowledge, occurs by passive diffusion [89,90]; however, since both actin and myosin are components of exported ribosomal subunits and ribonucleoprotein (RNP) particles, it has been assumed that they play some role in the ribosome and RNP transport within the nucleus and/or across the NPC. But our knowledge of this is still very limited.

An additional example of intranuclear motility is provided by the movement of viral particles in the nucleus. Previous data on this in the case of actin are related to the replication of baculoviruses, herpesviruses and human immunodeficiency virus type I [91], while among the myosins, supporting results have been described for myosin I and myosin Va [92]. Accordingly, in insect cells, inactivation of the Hsp90 chaperone prevented baculovirus-induced nuclear actin polymerization and the subsequent nuclear release of newly assembled nucleocapsids. The explanation for this phenomenon is that Hsp90 indirectly controls the protein levels and nuclear accumulation of the P40 subunit of the host ARP2/3 complex, which in turn regulates nuclear actin polymerization required for virus egress [93]. However, some recent results contradict the idea that the actomyosin machinery actively transports nucleocapsids within the nucleus. Actin has been detected in the RNP of the Epstein-Barr virus LMP2 protein mRNA, but associates with a structured intronic region of the LMP2 pre-mRNA, suggesting that nuclear actin may have a potentially new role in regulating viral RNA splicing rather than its transport [94]. Another study demonstrated physical association between the human cytomegalovirus (HCMV) UL53 protein, which regulates membrane fusion, and myosin Va in the nucleus, but contrary to reports regarding UL53 homologs in other herpesviruses, this interaction was not important for the migration of HCMV capsids toward the inner nuclear membrane [95]. Thus, the precise role of actin and myosins and their contribution to the intranuclear movement of viral particles is still a question. In addition, the possibility of a new, actin-based mechanism related to the entry of viruses into the nucleus has recently been raised. Baculoviruses utilize actin-based movement to travel from the cell periphery to the nucleus, and interestingly, it has been reported that actin polymerization is used by the cargo as a propulsive force to enter the cell nucleus through the NPC [96].

## Development

Understanding the molecular function of nuclear actin is important, but the biological significance of its activity and its consequences at the organ or organism level are perhaps even more important questions. Although the role of nuclear actin in stem cell differentiation and reprogramming has been studied since the beginning of nuclear actin research [97], the investigation of the physiological and developmental consequences of disrupting the function of nuclear actin has recently become intensive (Figure 1(g)).

Nuclear actin is essential for one of the most important aspects of development, the normal formation and functioning of germ cells. Actin filaments and bundles were found in the nucleus of prophase-arrested, healthy oocytes, and perturbations of this actin network were shown to restrict chromatin mobility and cause defects in chromosome alignment and segregation during meiosis [98]. Prostaglandin signaling was demonstrated to regulate nuclear actin during oocyte development in *Drosophila*. Loss of prostaglandin signaling increases G-actin levels in the nucleolus, and promotes the polymerization of nuclear actin during early stage oogenesis, concomitant with changes in nucleolar morphology and activity in nurse cells [99]. The Tootle lab has also found that the polymeric form of nuclear actin is also required for the maintenance of germline stem cells in *Drosophila* ovaries, as altering nuclear actin assembly leads to the loss of these cells and a progressive disturbance of the nuclear lamina, heterochromatin and nucleolus in their nuclei [100].

As with oocytes, nuclear actin has also been shown to be essential for the development of male gametes. The presence of DAAM in the sperm nucleus suggests the importance of actin polymerization [101]. Accordingly, nuclear F-actin and its polymerization dynamics have been found important in the regulation of the localization of IZUMO1, the key player in sperm-oocyte fusion, as well as chromatin remodeling and acetylation events in human spermatozoa [102]. In mice sperm cells, the proper remodeling of nuclear actin along the epididymal transit was shown to be dependent on circLIMA1 (a covalently closed RNA), possibly through the activation of gelsolin [103].

The nuclear activity of actin has been found to be crucial in various aspects of embryonic development following fertilization. Gradual nuclear actin accumulation and polymerization was observed in the nuclei of zebrafish embryos during early stage development. The accumulation was detected from interphase to prophase, and F-actin remained along the condensing chromosomes up until the prometaphase after nuclear envelope breakdown, suggesting its role in assisting chromosome congression and proper mitotic progression [104]. Decabromodiphenyl ethane, a flame retardant commonly found in electronic devices was shown to impact F-actin assembly in the mouse pronuclei (PN), resulting in the reduction of PN size and increased DNA damage, ultimately inhibiting embryonic development [105]. Zygotic development is a critical time of embryogenesis and the preservation of intact DNA is of utmost importance. In mouse zygotes induction of DNA damage by bleomycin causes the appearance of a pronounced nuclear F-actin network. Thwarting nuclear actin polymerization in zygotes leads to the accumulation of DNA damage and subsequent DNA damage checkpoint activation, resulting in the impairment of embryonic development. On the other hand, abnormal actin polymerization induced by the overexpression of actin-G15S (a polymerization enhancing mutant) in the nucleus alters genomic organization and also causes developmental arrest, highlighting that proper dynamics of actin polymer assembly and disassembly in the nucleus are indispensable for embryogenesis [106]. In human embryonic development, it was discovered that nuclear actin is crucial for the decidualization of the human endometrium. Tamura et al. observed that inducing decidualization with cAMP in human embryonic stem cells resulted in the assembly of a nuclear actin network, which is indispensable for the downregulation of cell proliferation markers to allow cells to exit from the cell cycle. C/EBPß was implicated in the process, possibly through the regulation of exportin-6 levels, however the exact molecular mechanism remains to be discovered [107].

The key role of nuclear actin in differentiation has long been observed [108]. Recently, it has also become clear that during muscle cell differentiation, not only sarcomere formation but also normal nuclear events require actin activity. ACTA2-coded smooth muscle α-actin (αSMA) was shown to localize to the nucleus of smooth muscle cells (SMC) and accumulate there throughout differentiation. αSMA is also involved in chromatin remodeling as part of the INO80 and BAF complexes, regulating the expression of SMC contractile genes during differentiation. R179 mutation of αSMA exhibited decreased nuclear localization, altering chromatin accessibility and promoting proliferation over differentiation [109]. Nuclear actin dynamics have also been implicated in vascular smooth muscle cell calcification. Stiffening of the extracellular matrix was shown to reduce nuclear G-actin levels, resulting in the promotion of osteogenic phenotype through YAP-mediated activation of TEAD and RUNX2 [110].

It has been earlier shown that nuclear actin is essential for regulating the fate commitment of mesenchymal stem cells (MSCs). The key diaphanous-related formin, mDia2, is found in the nucleus where it regulates actin polymerization, while mDia1 is responsible for controlling actin polymerization in the cytoplasm. The Rubin lab has shown that knock down of mDia2 causes loss in lamin B1 nuclear envelope structure and integrity, increased nuclear height, and reduced nuclear stiffness. This effect is facilitated by increased Runx2 transcription and strong osteogenic differentiation, as well as suppressed adipogenic differentiation [111]. A comprehensive study on the role of nuclear actin forms in EMT revealed that polymerized actin promotes EMT by interacting with β‐catenin, SMAD2, and SMAD3, while monomer actin represses EMT through MYBBP1A, NKRF and MYPOP [112]. Overexpression of hyperpolymerizing (S14C) or non-polymerizing (G13R, R62D) actin forms also increased the protein levels of their respective interacting partners in nuclei by enhancing their stability.

Nuclear shape affects cell migration, gene expression, and cell cycle progression, and it undergoes changes in conditions, such as laminopathies and cancer. Recent experiments with *Xenopus* egg extract suggested that nuclear F-actin and Lamin A antagonistically modulate nuclear shape. This could be confirmed in HeLa cells as well, and it was also revealed that in both frog and human cells this function of actin depends on formins and not on the ARP2/3 complex or myosins [113]. A novel role for nuclear actin in inhibiting cell proliferation and migration has recently been described. Increased levels of intranuclear actin monomer was found to inhibit cell proliferation and migration by suppressing MKL1-SRF and YAP/TAZ-TEAD-dependent gene expression. This mechanism mediates the anti-mitogenic and anti-migratory effects of physiological signals that elevate cyclic-AMP [114].

## Disease

There are many known human diseases related to actin, myosins, and other actin-binding and organizing proteins, but which of these conditions and to what extent they are due to the nuclear function of these proteins is still poorly understood. It has long been known that in cases of stress, neurodegenerative diseases or viral infections, a microfilament network or even actin rods appear in the nuclei of affected cells [115,116], but the molecular processes underlying this are not yet known.

In recent years, the role of nuclear actin in cancer has been the most studied of human diseases (Figure 1(h)). Since tumors often mimic developmental programs, which are regulated by mechanical factors, the implications of the nuclear localization of actin are closely associated with various characteristics of cancers. The polymerization of actin in the nucleus has been shown to be a key component for the metastatic capabilities of cancer cells. Recently, the O’Neill lab uncovered a molecular mechanism behind the metastatic ability of cancer cells and the alteration of nuclear actin levels. They found that in various mammalian cells, the microtubule associated protein, RASSF1A is localized to the inner nuclear membrane where it directly binds exportin-6. This interaction is mediated by a lamina-associated pool of the Hippo kinase, MST2, and is required for the association of RAN GTPase, actin, and profilin to the complex, as well as for the nuclear export of actin, which is crucial in regulating the serum response through the nucleocytoplasmic transport of MRTF-A. This pathway is aberrant in cancer cells where RASSF1A expression is lost and correlates with high levels of nuclear actin and reduced MRTF-A activity, leading to cell adhesion defects [84]. Given that epigenetic silencing of the RASSF1A gene is prevalent and linked to poor clinical outcomes in various solid tumors [117], this axis appears to play a functionally significant role.

In hepatocellular carcinoma cells (HCC), Huang et al. have shown that knockdown of the mitochondrial transcription factor TFAM resulted in the unexpected increase of polymerized nuclear actin [118]. A similar phenotype could be observed in mice and clinical samples of human HCC tissue. The importance of nuclear actin polymerization in the metastasis of HCC cells was highlighted by the fact that expression of non-polymerizable R62D actin in the nucleus led to a significant inhibition of the migration and invasion capability of tumorous cells. Nuclear actin polymerization was shown to be driven by the TFAM knockdown-induced malonylation and subsequent nuclear translocation of the mDia2 formin, and associated with a poor prognosis in HCC patients [118]. Nuclear F-actin structures have also been documented in ovarian cancer cells derived from patients. A deep learning approach identified the PI3K-AKT pathway as a potential regulator of nuclear actin polymerization in ovarian cancer [119]. According to a recent report, in triple-negative breast cancer stem cells lovastatin, an FDA-approved lipid-lowering drug, induces actin translocation into the nucleus and leads to nucleolar stress [120]. However, the underlying mechanisms and consequences remain to be explored. Interestingly, in mouse pancreatic adenocarcinoma cells, a decrease in nuclear actin levels was found to be necessary for metastatic ability. The hepatocyte growth factor (HGF), a ligand of the MET receptor tyrosine kinase, promotes the internalization and nuclear docking of another receptor tyrosine kinase, EphA2. This process depletes the nuclear G-actin pool by inducing cofilin phosphorylation, which reduces actin import. Additionally, juxta-nuclear actin polymerization further affects the availability of G-actin for nuclear import. Depletion of nuclear G-actin levels in turn enhances invasive behavior in cancer cells by activating the MRTF-A transcription factor [121]. The manipulation of nuclear actin has also been explored as a therapeutic approach, by taking advantage of the role of actin in DNA repair to enhance the tumor-killing effect of doxorubicin, a chemotherapeutic agent known to induce DNA DSBs. The global change of actin levels in the cell either by stimulating or inhibiting polymerization was shown to impair non-homologous end joining (in the case of F-actin dominance) and homology directed repair after inducing DSBs with doxorubicin, by suppressing the recruitment of repair factors to the damage site, thus enhancing the demise of tumor cells [40].

The formation of nuclear microfilaments has recently been linked to many other diseases besides cancer (Figure 1(h)). Nuclear actin was shown to be affected in Hutchinson-Gilford progeria syndrome (HGPS), as the disease is caused by the expression of a mutant form of lamin A, progerin, which lacks one of the actin-binding sites of the wild-type protein [122]. In fact, it was demonstrated that progerin expression decreases nuclear F-actin formation, contributing to the aberrant shape of HGPS cell nuclei and the misregulation of their gene expression, which can be at least partially rescued by the artificial increase of nuclear F-actin assembly [123]. The importance of nuclear actin has been highlighted in the context of injury as well. Le et al. have described that injured alveolar epithelial cells show an increased level of nuclear actin, and also revealed its interaction with the phosphorylated form of EZH2 and Pol2, forming an injury-induced fibrotic transcriptional complex [124]. A recent study confirmed that the polymerization status of nuclear actin also modulates immune function. Elevation of nuclear monomer actin levels mediated by increased cAMP signaling were associated with the repression of the TNFα-stimulated expression of inflammation-linked genes dependent on NF-κB, through promoting the proteasomal degradation of the NF-κB subunit RelA/p65 [125]. In ulcerative colitis, a chronic bowel disease, CircNlgn, a circular RNA and Nlgn173, the protein it encodes were found to be highly expressed in the colon of patients with colitis, promoting the progression of disease development. Nlgn173 was shown to translocate into the nucleus, where it binds actin and enhances its phosphorylation at Y53, thereby inhibiting actin polymerization by ARP2/3 in the nucleus [126].

Nuclear actin polymerization was also implicated in T cell function. T cell activation driven by T cell antigen receptor (TCR) engagement was shown to induce the transient formation of nuclear actin filaments mediated by ARP2/3. The transient burst of Ca^2+^ release upon activation was determined to be the triggering force behind nuclear actin polymerization, with critical involvement of calmodulin and the CaM kinase II [127]. Recently, the ARPC5L subunit of the ARP2/3 complex was found to be responsible for nuclear F-actin formation upon stimulation in CD4 + T cells, with no effect on cytoplasmic actin polymerization which is governed by another subunit of the complex, ARPC5. Interestingly, replication stress-induced nuclear F-actin formation was found to be mediated by ARPC5, independently from nuclear Ca^2+^ signaling [128]. The Chaperonin containing tailless complex polypeptide 1 (CCT) complex has been previously described as a chaperon for both actin and tubulin [129]. Loss of CCT complex member CCT8 in T-cells impaired the formation of nuclear actin filaments, but did not affect the expression of actin [130]. Titelbaum et al. provided evidence that nuclear F-actin in helper T cells colocalizes with chromatin fibers and EZH2, a component of the polycomb complex. Inhibition of the methyltransferase activity of EZH2 was shown to abolish the formation of nuclear actin filaments and also reduce the expression of ß- and γ-actin, suggesting that EZH2 regulates actin assembly transcriptionally and posttranscriptionally as well. The study also demonstrated that nuclear actin polymerization mediated by EZH2 is necessary for chromatin spreading and nuclear expansion during helper T cell differentiation [131].

## Methods

For a long time, the major obstacle to researching nuclear actin was clearly technical difficulties [32]. The biggest challenge from the beginning has been the nucleus-specific manipulation of actin without disrupting its essential cytoplasmic functions, a problem that has not yet been satisfactorily resolved. A recent attempt to specifically manipulate nuclear actin was to equip actin with a nuclear export signal to reduce nuclear levels of actin in *Drosophila* [87], but the method only caused a small reduction in nuclear actin levels without major physiological effects. A different type of approach for the future could be the spatiotemporally controlled perturbation of nuclear actin activity by cell-permeable, photoswitchable jasplakinolide [132,133] and latrunculin [134] small molecules developed recently, or by NLS-tagged synthetic bicyclic peptides which specifically bind to G-actin but not to F-actin [135].

Another problem in the field is the reliable visualization of actin. The usefulness of visualization tools, whether they are actin binding drugs or proteins or even labeled actin, is limited because they all affect the behavior and polymerization of actin, and therefore its function [42,136–138]. Although this is a general problem in actin research, it is not specific to the study of nuclear actin, but because of these limitations, actin probes should always be carefully titrated to minimize the risk of artifacts. Adding to this problem is the fact that actin can also occur in the nucleus in many different forms, which may require different methods to visualize it. Shindo et al. have described an optimized staining method for the visualization of endogenous nuclear F-actin in mouse embryos. Using their new staining method, the authors have discovered that somatic cell nuclear transferred embryos exhibit abnormal nuclear actin structure, showing more short filaments as opposed to long ones, compared to *in vitro* fertilized zygotes, highlighting an important difference between fertilization and nuclear transfer techniques for embryo production [139]. For imaging stress-induced intranuclear actin rods in live *Drosophila* embryos, Biel and colleagues devised a protocol by mounting the embryos on a coverslip followed by injection with rhodamine-conjugated G-actin and subsequent microscopy [140]. Live cell imaging of nuclear actin filaments by expressing EGFP and NLS-tagged actin chromobody in cultured insect and mammalian cells has also been used to study the spatial and temporal dynamics of heterochromatin repair foci, which cannot be accomplished with fixed cell studies or biochemical approaches [141].

Fluorescent labeling of actin protein has also been a long-standing tool for visualizing actin structures, and the consensus is that N-terminal labeling interferes less with the functions of the molecule. Cell lines that produce *in situ* tagged actin using CRISPR are now available. This approach, while convenient, does not eliminate the potential side effects of tagging, but it does address one problem, as the production of the protein ensures the wild-type state [142–144]. The Uyeda lab recently expressed an N-terminally labeled actin, β-actin-EGFP fusion protein, in U2OS cells and observed that it displayed a monomeric to filamentous ratio similar to that of endogenous actin, unlike the commonly used EGFP-β-actin fusion protein, which tends to over-assemble. Interestingly, curved bundles of β-actin-EGFP were formed in the nucleus and they were not labeled with rhodamine phalloidin, Lifeact-EGFP or anti-actin antibodies, and were rich in cofilin [145]. Based on all this, the authors speculate that posttranslational modification of the N-terminus of actin may be required for nuclear translocation or for the formation of these intranuclear actin structures. By comparing multiple labeling tools, they also found that the expression of nuclear actin probes affected the formation of actin filament structures. NLS tagged GFP-based probes for actin, such as EGFP-NLS-β-actin, Lifeact-NLS-EGFP or actin chromobody visualized thin actin filaments and some aggregates in the nucleus. In contrast, skeletal α-actin (V163M-EGFP) formed thick, arc-shaped structure in the nucleus, and the expression of the actin binding domain of supervillin (EGFP-SVIL) induced the formation of thick, meandering bundles of nuclear actin filaments [145]. Despite the difficulties and unresolved problems mentioned above, the application of genomic methods has gained popularity in the study of nuclear actin in the past few years. The possibility of studying changes in the organization and function of the genome as a consequence of nuclear-specific perturbations of actin dynamics and activity has clearly opened a new dimension in the field [17,20,49,50,76,114].

## Discussion

The exploration of actin’s nuclear activity is certainly one of the most exciting new perspectives in cell biology and actin research today. The results so far and those to be expected go far beyond actin, as they will also provide many valuable and interesting insights into human diseases, the functioning of the nucleus and even the evolution of eukaryotes. However, these studies are hindered by the abundant presence of the protein and its extremely versatile, essential functions in the cytoplasm, which is why the reliable separation of actin’s cytoplasmic and nuclear functions is still difficult. Although actin visualization methods are constantly offering new possibilities, and there are promising attempts to manipulate actin in space and time, the past 5 years have not brought any real breakthrough in this regard. Fortunately, continuous advances in laboratory tools and techniques, most recently super-resolution microscopy and genomic methods, have facilitated the elucidation of more and more details of actin’s functioning in the nucleus and have already helped us to achieve significant progress.

Thus, over the past few years it has become clear that the polymeric form of actin performs much more important tasks in the nucleus than previously thought. Direct data have demonstrated that F-actin plays role, for example, in the integration of gene expression-stimulating effects, the mobilization of replication error repair factors, and the movement of chromatin. Recent data have also revealed a lot about the details of these activities of nuclear actin. Although direct confirmation is still awaited, it can now be stated with a fair degree of certainty that actin microfilaments are also essential for the formation of nuclear factories, as they appear to provide the basis for the phase separation process necessary for nuclear body formation. In addition, recent data show that actin-binding proteins surprisingly often have disordered regions, which further strengthens the idea that nuclear actin is a key player in the separation of liquid–liquid phases. An important task for the future is to clarify how actin participates in nuclear LLPS and how essential its role is in the process.

From the beginning of nuclear actin research, the emphasis has been on the exploration of its monomeric functions. Although, the focus of studies has recently clearly shifted to the function of polymeric actin, research on G-actin, for example in histone modifying and ATP-dependent remodeling complexes responsible for genome rearrangements, remained intensive. The 3D structure data that have been published in the past few years have provided very important details and helped us to come close to understanding the precise role of actin in chromatin remodeling.

The above-mentioned nuclear processes and the phase-separated nuclear bodies are essential for the basic functions of the cell nucleus, and thus it becomes understandable why the presence of actin in the nucleus is over-secured and dynamically regulated. We know relatively much about the nuclear transport of actin, but recent new results on the viral transport within the nucleus, the parallel nuclear import pathways of actin and its evolutionary differences have highlighted that the nuclear transport processes related to actin are much more complex and intricate than previously thought. Clarifying this and unraveling the details is another important and major task for the future.

## Data Availability

Data sharing is not applicable to this article as no new data were created or analyzed in this study.
